# Chikungunya virus: Molecular epidemiology of nonstructural proteins in Pakistan

**DOI:** 10.1371/journal.pone.0260424

**Published:** 2021-12-23

**Authors:** Nazish Badar, Aamer Ikram, Muhammad Salman, Muhammad Masroor Alam, Massab Umair, Yasir Arshad, Nighat Mushtaq, Hamza Ahmad Mirza, Abdul Ahad, Umer Farooq, Muhammad Talha Yasin, Javaria Qazi

**Affiliations:** 1 Department of Biotechnology, Quaid-I-Azam University, Islamabad, Pakistan; 2 Department of Virology, National Institute of Health, Chak Shahzad, Islamabad, Pakistan; 3 National Agricultural Research Center, Chak Shahzad, Islamabad, Pakistan; Ghazi University Dera Ghazi Khan, PAKISTAN

## Abstract

Chikungunya virus (CHIKV) is considered a public health problem due to its rapid spread and high morbidity. In 2016–2017 an outbreak of CHIKV was occurred in Pakistan but the data regarding the genomic diversity of CHIKV was not reported. Hence, the current study aimed to determine the genetic diversity of CHIKVs in Pakistan. A cross sectional study was carried out using sera of infected CHIKV patients (n = 1549) during the outbreak in Pakistan (2016–2018). Nucleotide sequencing of non-structural genes of CHIKV from eight isolates were performed followed by phylogenetic analysis using Bayesian method. Phylogenetic analysis suggested that the Pakistani CHIKV strains belonged to Indian Ocean Lineage (IOL) of genotype ECSA and C1.3a clade. Furthermore, the Pakistani isolates showed several key mutations (nsP2-H130Y, nsP2-E145D, nsP4-S55N and nsP4- R85G) corresponding to mutations reported in 2016 Indian strains of CHIKV. The molecular analysis revealed high evolutionary potential of CHIKV strains as well as better understanding of enhanced virulence and pathogenesis of this outbreak. The study highlights the need to continue surveillance in order to understand viral diversity over time and to devise preventive measures to limit diseases transmission in the region.

## Introduction

Increased virulence of emerging and re-emerging pathogens, like Chikungunya (CHIKV), Dengue (DENV) and Zika (ZIKV) viruses is of serious public health concern [1–3]. Chikungunya (CHIK) is zoonotic viral infection transmitted by the bite of female mosquito of *Aedes* family (*Ae*. *aegypti* and *Ae*. *albopictus* species). The case fatality rate is negligible. However, the clinical manifestation includes high fever, headache, myalgia accompanied by chronic discomfort (rheumatic pain, fatigue, depression) and skin rashes. The debilitating nature of this disease leads to outbreaks of considerable economic burden and can breakdown health systems [4–6]. The clinical presentation of CHIK resembles with other viral diseases, like Dengue and Zika as they share same vectors for transmission. Hence, it is not possible to differentiate between CHIK, DENV and ZIKV infections without laboratory diagnosis [7, 8].

CHIKV is RNA alphavirus that belongs to *Togaviridae* family [1]. The 12 kb CHIKV genome comprises four nonstructural proteins (nsP1, nsP2, nsP3 & nsP4), a core protein, three envelop proteins (E1, E2, E3), and short 6K region. Several gene mutations are reported in structural and nonstructural proteins which are associated with virulence and infectivity of the virus [9–11]. The RNA is translated directly into the cytoplasm after the entry and starts replication. The nonstructural proteins (NsPs) of CHIK virus are encoded by the 5′ region of viral RNA genome. Although the replicative functions of CHIKV NsPs are unknown but some of the NsPs (name of Nsps) were fully characterized to differentiate from other alphaviruses like, Semliki Forest virus (SFV) and Sindbis virus [12]. Overall, the biochemical functions are conserved in CHIKV, SFV, and Sindbis virus proteins but the amino acid sequence is 71% identical with SFV NsPs.

The CHIKV has a single serotype and three genotypes, associated with spatial and temporal distributions of mosquito vector (*Aedes* mosquito species). Phylogenetic analysis showed three lineages of CHIKV: the Asian (AS), West African (WA) and East/Central/South African (ECSA) within Indian Ocean (IO) lineage [4]. Furthermore the ECSA lineage is sub divided into two clades: ECSA1, comprising ancestral CHIKV sequences and ECSA2 which contain sequences from Central African Republic, Cameroon, Gabon, and Republic of the Congo [13].

CHIKV was first discovered in Tanzania in 1953 and later in Asia in 1958. Since then, various outbreaks and sporadic cases were reported during 1960s and 1990s which were limited to Africa and Asia [14]. However, during 2005 CHIKV disease re-emerged and spread to the Indian Ocean islands and India to South East Asia causing severe outbreaks with high mortality. Till now more than 100 countries reported local transmission of CHIKV in Asia, Africa, Europe, and the Americas [15].

In Pakistan the first case of CHIKV was reported in 1983 in rodents and later in 2011 the virus was reported in Humans during the dengue outbreak. As the signs and symptoms of CHIK and dengue disease resemble so the CHIK may be misdiagnosed as Dengue fever.

After 2011 no case of CHIKV was reported from Pakistan. [8]. The first case of CHIK was again reported in December, 2016 from Karachi. This would lead to an outbreak of CHIK in Sindh and KPK with 2582 confirmed cases [16]. The widespread occurrence of *Aedes* species across Pakistan resulted in local transmission of CHIK and an outbreak of 2016–2017.

Despite an outbreak of CHIK in Pakistan, the knowledge about prevalent genotypes of CHIKV was not available. Hence, in-order to identify the prevalent genotype of CHIKV in Pakistan during 2016–2017 outbreaks, the current study was carried out. The identification of circulating lineages in Pakistani population will help to understand the diversity of CHIKV in Pakistan. This will further help to devise strategies to stop future outbreaks.

## Material and methods

### Case definition

The World Health Organization’s definition for chikungunya standardized case was used to screen the cases, based on following clinical and epidemiological features: acute onset of fever >38.5°C lasting for 7 days, one or more symptoms: nausea, diarrhea, vomiting, eye pain, hemorrhagic manifestation, rashes, joint pain, swelling in joints, severe arthritis [17].

### Study population

Blood samples were collected from suspected chikungunya patients between 20^th^ December, 2016 to July, 2018 from different hospitals and outdoor clinics in various geographic regions in the country across Pakistan. Blood was collected in heparinized collection tubes and centrifuged at 2000 rpm for 5 minutes for serum isolation which was stored at -80°C until further use. A predesigned questionnaire (patient medical record) including epidemiological information (demographic, clinical characteristics, travel history and risk factors for exposure to mosquito bites) was filled at the time of sample collection.

### Ethical clearance

National Institute of Health Pakistan Ethical Committee approved the study containing standard guidelines for surveillance and sampling protocols. Written consent was also obtained from patients.

### Laboratory diagnosis using real-time PCR

Nucleic acid was extracted from serum using QIAmp viral RNA minikit (Qiagen, GmbH) according to manufacturer’s protocol and eluted in 60μl elution buffer. All samples were processed using CDC Trioplex real-time RT-PCR (DENV, CHIKV & ZIKV) one-step real-time reverse transcription-polymerase chain reaction (rRT-PCR) assay on Applied Biosystems platform (ABI7500) [18]. Briefly, SuperScript Platinum III One-Step RT-PCR Kit (Life Technologies) was used for the PCR reactions. A final volume of 15μl PCR reaction mix for multiplex assay containing 0.5μl of each probe including: dengue (FAM labeled), chikungunya (VIC labeled) and Zika (Texas Red labeled), 0.5μl of forward and reverse primers, 12.5ul of 2X master mix, 0.5 μl enzyme mix, and 0.5μl nuclease-free water. Rnase-P (FAM Labelled) was run with every assay as an internal control and 10μl of extracted RNA was used for amplification with cycler conditions; reverse transcription for 30 min at 50°C, *Taq* polymerase activation for 2 min at 95°C and 45 cycles for 15s at 95°C with an extension step of 1 min at 60°C.

### RT-PCR for CHIKV

Eight CHIKV isolates (two from each province) were randomly selected for complete CHIKV nonstructural protein coding nucleotide sequences from different geographic regions namely Sindh, Baluchistan KPK and Punjab.

Specific CHIKV oligonucleotide primers [4] were used for RT-PCR/sequencing of complete nonstructural genes (NsP1, NsP2, NsP3, and NsP4) from prototype African strain S-27 as summarized in S1 Table. The reverse transcription RT-PCR assay was carried out using Qaigen one Step RT-PCR kit (GmbH) in a total 50μl volume with 20 μmol each of forward and reverse primers and 3 μl of the extracted RNA [19]. PCR reactions were conducted on a Veriti PCR thermal cycler (Applied Biosystems, USA) using the protocol; 30 min at 42°C for reverse transcription and 3 min at 94°C for Taq polymerase activation; 35 cycles of for 45 sec at 94°C, 1 min at 54°C, 1 min at 60°C and final extension step for 5 min at 60°C. Amplified products were visualized on 1.5% agarose gel.

### Nucleotide sequencing

The RT-PCR products were purified using Axy Prep ^TM^ Mag PCR (Axygen) clean up kit, according to the manufacturer’s protocol. The PCR products were sequenced using sequencing primers both in forward and reverse directions on an ABI Prism 3130 (Applied Bio Systems, USA) using an ABI Prism BigDye Terminator cycle sequencing kit v3.1 (Applied Biosystems, USA).

### Sequences and dataset construction

Data set was constructed using eight sequences from Pakistan along with CHIKV sequences deposited in GenBank. Information regarding collection dates and locations were also gathered. In total, 65 sequences from different countries were used for phylogenetics. Sequence data were compiled and Contig sequences assembled using the DNA STAR Lasergene Package Version 6. Nucleotide sequences were aligned using ClustalW for CHIKV complete nonstructural region with MEGA version 6 [20, 21].

### Phylogenetic analysis

A time reversible gamma distribution (GTR+G) model was used by jModelTest [22] as the best-fit model for phylogenetic analysis with non-structural protein dataset. The Bayesian Markov Chain Monte Carlo (MCMC) method available in the BEAST v2.3.0 package [23] was used for estimation of substitution rate, divergence time and a strict clock model. BEAST analysis was run for 40 million generations to attain convergence of parameters by calculating effective sample size (ESS > 200) by TRACER version 1.6 (http://tree.bio.ed.ac.uk/software/tracer/). The maximum clade credibility (MCC) tree was calculated using Tree Annotator within the BEAST and visualized via Figtree v1.4.3.

### Statistical analysis

SPSS was used to enter and analyze the data (IBM, version 22). Geographic distribution of number of Chikungunya (CHIKV) cases were shown in density map that was constructed in ARC GIS software version 10.6.1. The age stratified data of CHIKV is shown in graph the correlation of the clinical symptoms of CHIKV positive and negative cases was investigated and presented in graph.

## Results

**Fig 1:**
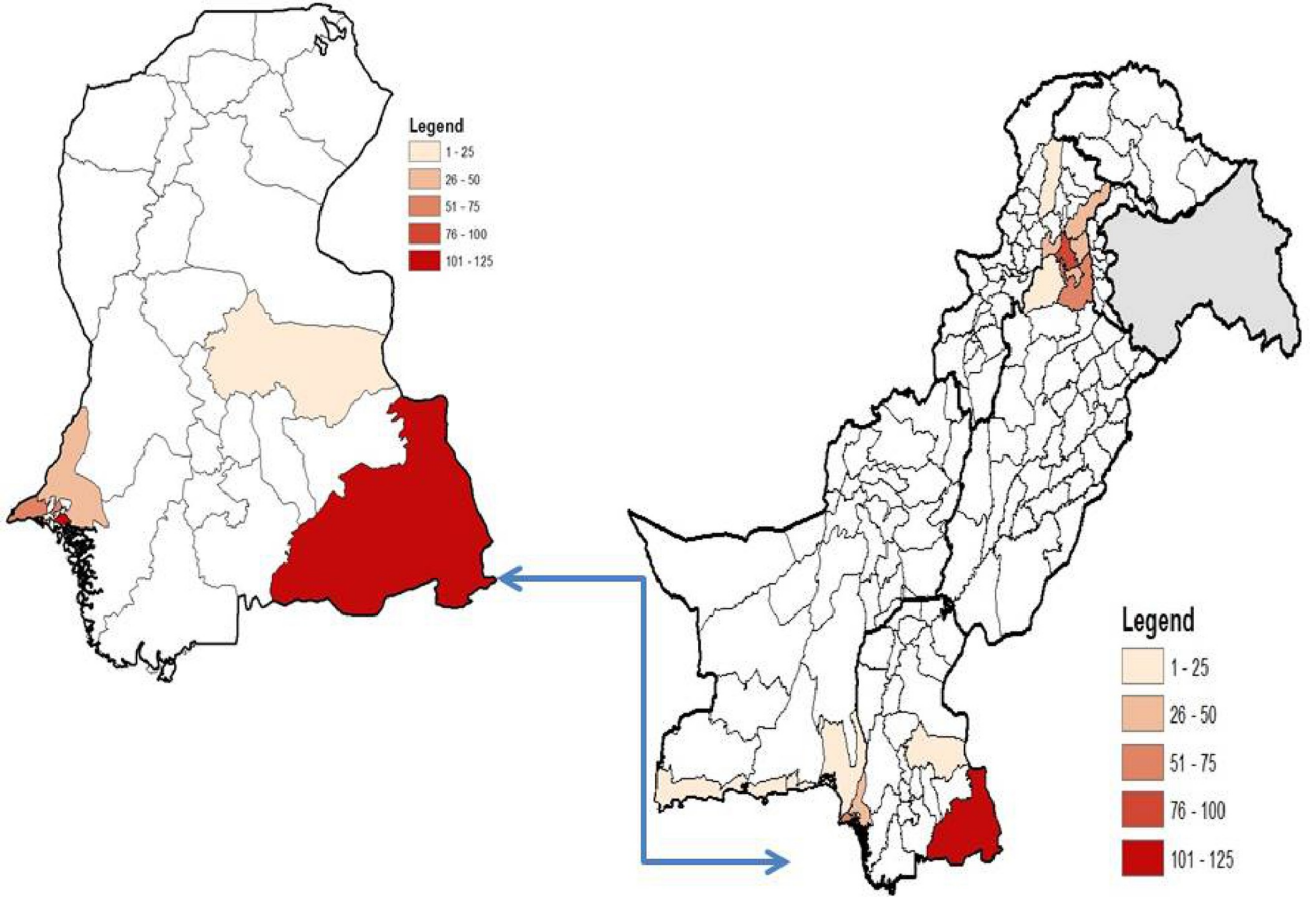
Geographic mapping of CHIKV positive cases from Dec, 16 to May, 18 in Pakistan.

**Fig 2:**
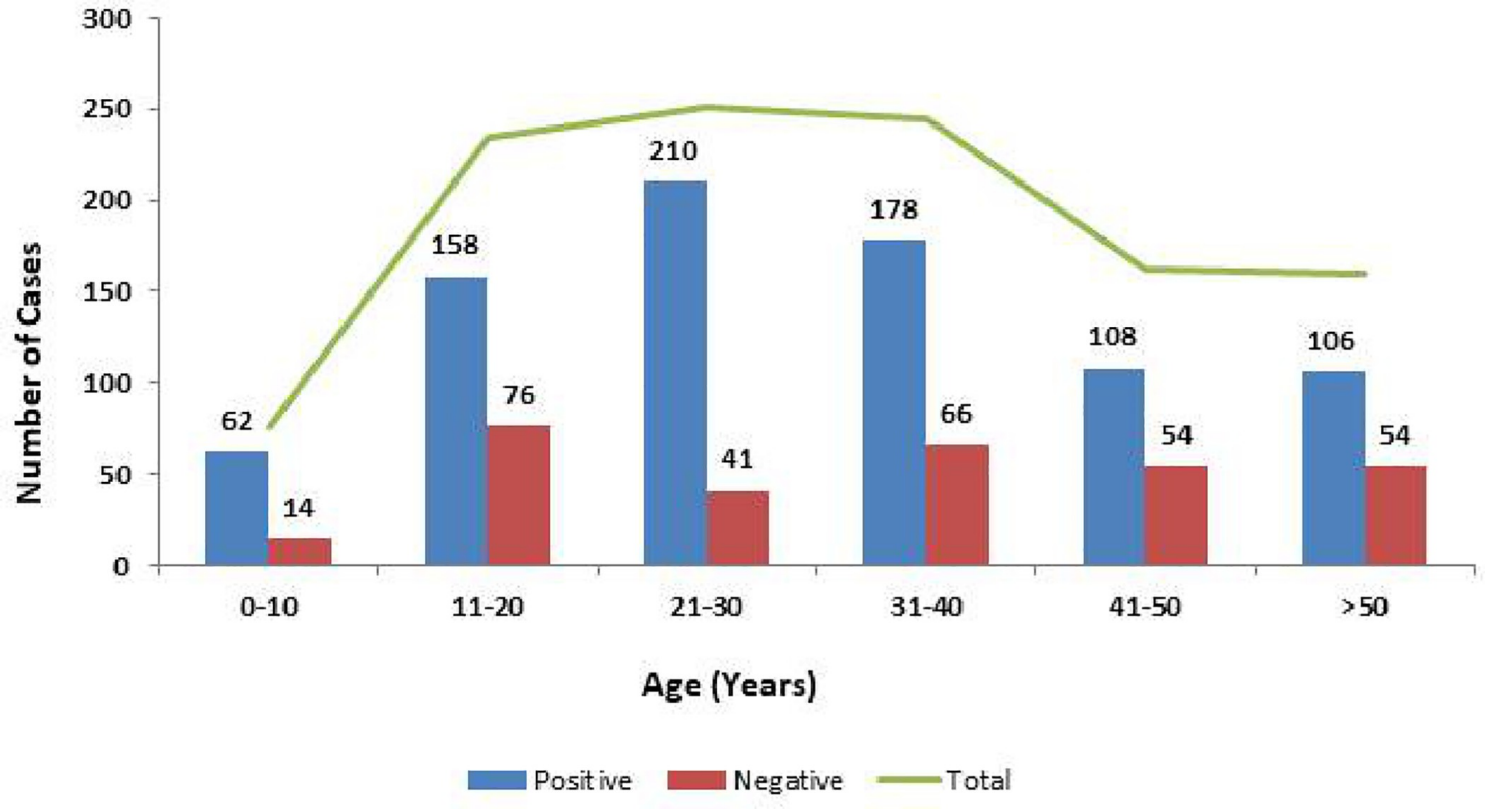
Age group distribution of Chikungunya positive and negative cases.

**Fig 3:**
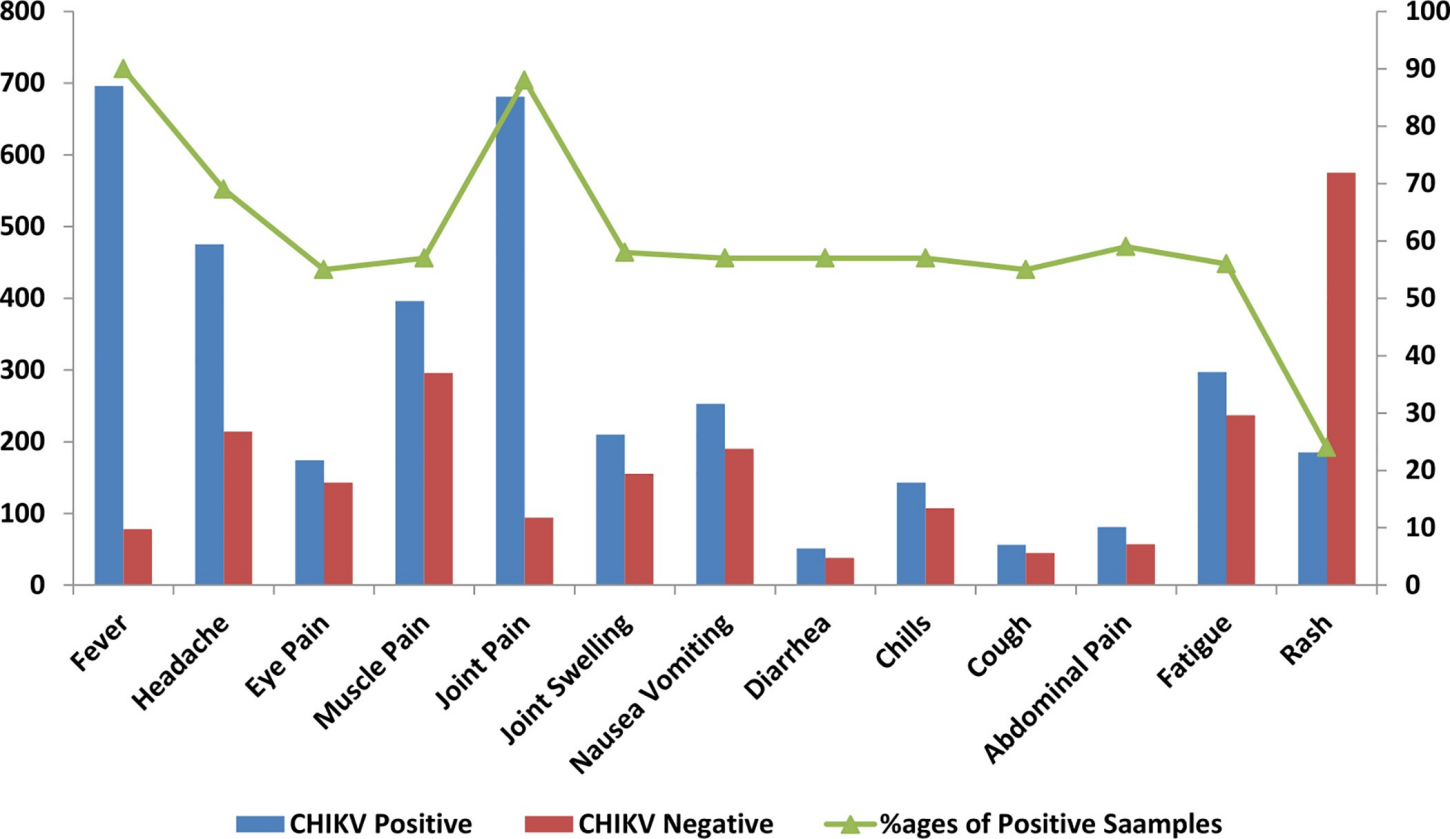
Association between Clinical Symptoms and Chikungunya virus.

A total of 1549 blood samples from suspected cases were processed by Real-Time PCR at virology department, National Institute of health Pakistan during 20^th^ December, 2016 to July, 2018. Out of which 50% (774/1549) were found positive for Chikungunya virus. The province wise distribution was as follows, Sindh: 474 (61.2%), Baluchistan: 30 (3.8%), federal capital: 28 (3.6%), Punjab: 54 (6.9%) and Khyber Pakhtunkhwa (KPK): 188 (24.2%). Geographic mapping of chikungunya positive cases in the country is presented in Fig 1. The mean age of Chikungunya positive patients was 31.8±15.7 years and the most affected age group was between 21 to 30 years (Fig 2). Fever (n = 696, 90%), headache (n = 534, 69%), joint pain (n = 681, 88%) and rash (n = 185, 24%) were the most common clinical symptoms (Fig 3). Children ≤10 years showed rashes (n = 325, 42%) compared to adult population (n = 449, 58%). Joint pain (91%) was observed in 11–20 years of age group and swelling was observed (40%) in 21–30 age group.

To explore the genetic diversity of CHIKV in Pakistan, eight CHIK virus isolates nonstructural genes were sequenced between 2016 (n  =  02) to 2017 (n = 06) and phylogenetic analysis was performed. Nucleotide and amino acid sequence data of Pakistani CHIKV strains was compared with sequences from various countries available in GenBank.

The complete nonstructural genome of eight CHIKV isolates from various geographic locations within Pakistan was generated corresponding to reference Tanzanian isolate S27 (AF369024.2).

Nonstructural gene sequences consisted of 7,422 nucleotides encoding 2,474 amino acids. The nonstructural polyprotein consists of nsP1 (535 aa), nsP2 (798 aa), nsP3 (530 aa), and nsP4 (611 aa) plays an important role in the viral RNA replication and translation.

### Phylogenetic analysis

Chikungunya strains detected in Pakistan showed >99% sequence similarity to the regional viruses reported from India, Bangladesh, and Srilanka and these clustered within the Indian Ocean lineage ECSA (East/Central/South African). The percentage divergence from the prototype Tanzanian isolate S27 at the amino acid level was 0.55 % to 1.1% and among Pakistani isolates.

Comparison of the sequences of Pakistani strains with ECSA Indian Ocean outbreak isolate revealed nine nucleotide substitutions (Table 1). Pairwise comparison of nucleotide sequences indicated a high similarity (99%) among Pakistani isolates. Comparison of 2016–2017 Pakistani strains with the S-27 African prototype strain showed 2.7% amino acid substitutions in 2017 isolates so these were clustered as separate group in the phylogenetic tree.

**Table 1:** Lineage-specific mutations of CHIKV from ECSA Africa and ECSA IOL comparing to Pakistan strains.

Protein		Non- Structural Polyprotein (2474)		
	NsP1	NsP2	NsP3	NsP4
	(535 aa)	(798 aa)	(530 aa)	(611 aa)
ECSA Africa	128T/K, 172L/V,	54S/N,	175V/I, 208T/I, 209K/R, 211Q/L,	55S/N,
	234E/K, 376T/M,	130H/Y,145E/D,	215Q/R, 228T/M, 231F/S, 253M/T,	76T/A, 85R/G
	383M/L, 384I/L,	374H/Y, 642C/Y,	258R/P, 264S/F, 268P/L, 270H/L,	500Q/L
	481T/I, 4588Q/R,	643S/N, 793A/V	274L/S, 275P/L, 276D/G, 281M/T,	
	507L/R		293E/S, 307P/L, 325T/I, 332Y/H,	
			334K/R, 337R/Q, 338P/S, 344A/V,	
			347I/T, 351T/M, 371P/H, 377Y/H,	
			382L/P, 415A/V, 421I/T,437Q/R,	
			449L/Q, 455R/H, 463V/I, 464T/EI,	
			470V/E, 478S/L, 503P/Q, 508I/T,	
			511I/T	
**Number of amino acid**	**9**	**7**	**41**	**4**
**Differences**				
ECSA IOL	476P/Q	21V/M, 136V/A,	378T/I, 484T/K, 539L/S	82R/S, 585M/T
		666Y/H		
**Number of amino acid**	**1**	**3**	**3**	**2**
**Differences**				
**Number of percentage**	1.9	1.2	8.3	1
**Differences %**				

**Fig 4:**
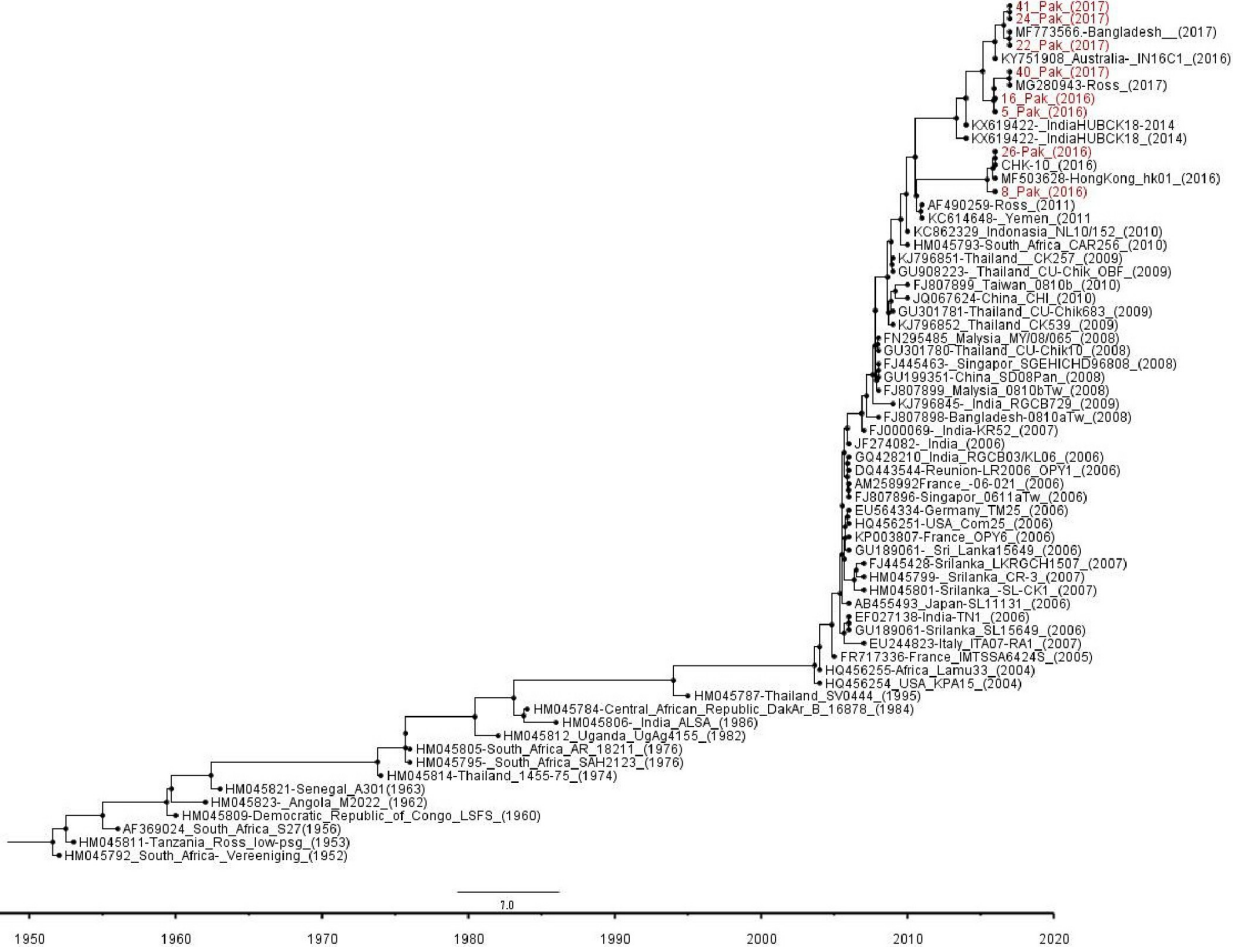
Phylogenetic tree of Chikungunya viruses generated by BEAST method on the complete Non-structural genes (NsP1, NsP2, NsP3 and NsP4) nucleotide sequence.

Phylogenetic analysis revealed that the CHIKV strains further divided into three lineages starting from Africa and Asia (Fig 4). The Democratic of Congo 2000 isolates clustered within the Indian Ocean Lineage ECSA. Asian and West-African isolates were far related to Indian Ocean lineage and formed a separate group. The prototype African S-27 strain was positioned at the root of the chikungunya isolates.

Indian Ocean lineage is further divided into three clades, analysis showed that Pakistan viruses belongs to clade C2.3a which are closely related to 2016 strains of India, Bangladesh and Thailand. These sequences showed five distinct substitutions: two in NsP2: H130Y, E145D, two in NsP4: S55N, R85G and one in Nsp3 at position D372E in amino acid sequence. However, an additional substitution was also observed in Bangladeshi strains 2017 in NsP2 at position 793 V/A.

### Amino acid sequence variations

Amino acid sequence analysis of the nonstructural genes (NsPs 1–4) of Pakistani isolates during 2016–17 were compared with prototype Tanzanian strain S27 that showed mutations at specific positions. Overall, the nonstructural proteins of isolates showed 70 (2.8%) aa substitutions (Table 1). NsP1 protein showed 10 (1.9%) aa changes, NsP2 showed 10 (1.2%), the NsP3 showed higher mutations 44 (8.3%) and NsP4 had 6 (1%) amino acid changes.

Amino acid sequence analyses of the nonstructural genes of Pakistani isolates during 2016–2017 were compared with global isolates. Sequence analysis of 2016 isolates showed three additional substitutions: one at NsP2 position Y666H and two in NsP3 gene at position 432 G/E and T484K when compared to 2017. However, 2017 isolates had one additional substitution in NsP2 gene at position 671 V/A then 2016 outbreak viruses.

Ten amino acid positions in the nonstructural proteins (Table 2) that were distinctive in the study isolates: Two mutations were seen at the conserved positions in Nsp2 at position 54, 374, two in NsP4 254 and 500. The remaining six changes were found in other regions.

**Table 2:** Aminoacid changes of CHIKV from ECSA Africa and ECSA IOL comparing to Pakistan strains.

Protein					Nonstructural Proteins					
	NS1	NS2	NS2	NS2	NS2	NS2	NS3	NS3	NS4	NS4
Polyprotein Position	**128**	**589**	**665**	**680**	**909**	**1328**	**1391**	**1705**	**1918**	**1948**
Protein Position	**128**	**54**	**130**	**145**	**374**	**793**	**58**	**372**	**55**	**85**
CHIKV-Pak-5-2016	K	N	Y	D	Y	V	V	D	N	G
CHIKV- Pak-8-2016	K	N	Y	D	Y	V	V	D	N	G
CHIKV- Pak-26-2016	K	N	Y	D	Y	V	V	D	N	G
CHIKV- Pak-10-2017	K	N	Y	D	Y	V	V	D	N	G
CHIKV- Pak-16-2017	K	N	Y	D	Y	V	V	D	N	G
CHIKV- Pak-22-2017	K	N	Y	D	Y	V	V	D	N	G
CHIKV- Pak-40-2017	K	N	Y	D	Y	V	V	D	N	G
CHIKV- Pak-41-2017	K	N	Y	D	Y	V	V	D	N	G
S27	T	S	H	E	H	A	V	D	S	R
Ross River	T	S	H	E	Y	V	V	D	S	R
Thailand	K	N	H	E	Y	V	V	D	N	G
Central African region	T	S	H	E	Y	A	V	D	S	R
Tanzania	K	N	H	E	H	A	V	D	S	R
Bangladesh	K	N	H	E	Y	V	V	E	N	G

### Glycosylation sites of nonstructural proteins

N-glycosylation sites in the nonstructural proteins were analyzed in study isolates in comparison with the prototype African strain S-27. N-glycosylation sites in NsP1, NsP2 NsP3 and NsP4 were found conserved. A single potential glycosylation site was reported in the NsP1 molecule of the South African strain S-27 virus at position 111. However, one site in NsP2 protein at position 775, four sites reported in NsP3 protein at position 72, 75, 241, 544 and three sites in NsP4 at position 62, 210, 445 in reference to South African strain S-27. All these glycosylation sites were conserved in all Pakistani isolates.

## Discussion

Chikungunya fever is an emerging arbovirus infection, representing a worldwide health problem. Pakistan like other countries is facing considerable environmental changes, the rise in temperature has provided suitable flourishing environment to viruses that caused arboviral illness such as dengue and malaria in the region. Asian countries provide suitable breeding grounds for arboviruses and arthropod vectors owing to the poor sanitary conditions that intensify the conditions. Karachi is a provincial capital of Sindh, Pakistan, largest coastal city that observed CHIKV outbreak in 2016 and 2017. Afterwards in 2017–2018, CHIKV infection was continued to spread in nearby areas and CHIKV cases surprisingly upsurge to a significant number. The infection was mainly observed in travelers and residents of Punjab and KPK. The confirmed CHIKV cases were 7, 762 and 5 in 2016, 2017, and 2018, respectively reported by National Institute of Health [24].

The morbidity rate of CHIKV and severity of illness varied at the geographic locations during the outbreak that involved hilly areas versus the coastal area and presence of Ae. albopictus mosquitoes during 2017 and 2018. The clinical manifestations observed during the outbreak were joint pain found in 73% patients, painful arthralgia that persists for a long time period [16]. A major difference observed in the clinical manifestations during the outbreak was skin rashes that are different from other global CHIKV outbreaks. Kannan et al., [25] observed these symptoms in 80.8% patients during CHIKV outbreak of 2007–2008 in Kerala, India. Similar observations were made by Talarmin during Re’Union Island outbreak who found that 19.3% patients have symptoms similar to other CHIKV outbreaks [26]. 75% CHIKV infected children were reported with predominant peripheral cyanosis, skin and dermatological manifestations such as skin peeling, maculopapular rash, erythema or vesiculobullous lesions [27]. The severity of CHIKV infections could be linked to genetic variations in the CHIKV genome [4, 28, 29]. To identify the possible causes of severity in CHIKV infections, we sequenced and analyzed the variations in non-structural gene of CHIKV isolates obtained during the outbreak. The study found that different genetic heterogeneity exists in non-structural genes of viral CHIIKV genome scattered in Pakistan in comparison to earlier studies [30].

Phylogenetic analysis showed a closer association among 2016 and 2017 isolates that belong to distant geographic locations. These results suggested that CHIKV strains that resulted in the later outbreaks of 2017–18 were found similar to those strains observed in outbreak of 2016 [31]. The nonstructural protein nsP2 reportedly contributing towards viral cyto pathogenicity of these CHIKV strains [11, 32, 33]. Pak CHIKV isolates contain amino acid variations in the non-structural protein at Viral Helicase domain. S54 N and H374Y were the two significant mutations observed in viral helicase domain [34, 35]. Sequence analysis showed the nsP4-R82S mutation in CHIKV is previously reported in Southeast Asia. This is a naturally occurring nsP mutation in alphaviruses which increases human host replication rather than Mosquito vector, which may lead to higher viraemia and more efficient human-mosquito transmission, thus contributing to extensive spread in the region [36, 37]. Pakistani viruses also showed this mutation in 2016–17 isolates. *Ae*. *aegypti and A*. *albopictus* were the mosquito species responsible for transmitting the CHIKV to humans [38]. The lineage analysis suggested the evolution of CHIKV isolates from Pakistan during 2016. The mutations reported in Viral helicase domain of non-structural protein involved in RNA replication in emerging CHIKV strains would contribute in understanding the function of viral non-structural proteins, genotype distribution and host specificity. This study reporting non-structural protein sequence analysis of CHIKV strains isolated during the 2016–18 outbreak in Pakistan. Further analysis revealed that there was high sequence similarity of Pakistani strains to those found in ECSA.IOL lineage. *A*. *albopictus and A*. *agyptii* were considered to transmit ECSA.IOL lineage, extensively scattered and distributed in sub-continent region [39–43]. It is suggested that the CHIKV strains were present and circulating in Pakistan but neglected owing to insufficient diagnostic facilities. The Pakistani residents are still at risk to be exposed to CHIKV infection.

These findings presented here would contribute towards understanding the transmission and evolution pattern of CHIKV for better epidemiological analysis and helps the higher authorities to prevent and control the spread of disease. Vector control is the only way available at present to curb the CHIKV transmission due to absence of effective anti-viral therapy and unavailability of vaccine. However, adaptation to different environments by *A*. *albopictus* and *A*. *aegypti* vectors may allow them to expand in new areas [44]. The molecular data presented in this study on outbreak clinical isolates will contribute to fill the knowledge gaps to better understand pathogenicity of CHIKV and will help to provide more reliable baseline information which will serve as a powerful tool to combat it.

In conclusion, the current study identified the prevalent genotype of CHIKV during 2016–2017 outbreaks. This is the first report about genotype analysis of CHIKV from Pakistan. Moreover, the current study also highlights the important mutations in the non-structural genes of CHIKV. The genotype data presented in the study will help to devise strategies about the containment of future outbreaks of CHIKV in Pakistan.

## Supporting information

S1 Table Nucleotide sequences of conventional RT-PCR/sequencing primers for CHIKV non- structural genes (NsP1, NsP2, NsP3 and NsP4). (DOCX)
