# Supplementary material for: Chikungunya virus: Molecular epidemiology of nonstructural proteins in Pakistan
Source: PLoS One. 2021 Dec 23;16(12):e0260424. doi: 10.1371/journal.pone.0260424 (PMC8699639; doi:10.1371/journal.pone.0260424)
Supplement: S1 Table — (DOCX) [file pone.0260424.s001.docx]

| Fragment | Gene | Primer (a) | Sequence (5' to 3') | Reference |
| --- | --- | --- | --- | --- |
|  |  |  |  |  |
| FG 1 | 5'NC | 18F | CACGTAGCCTACCAGTTTCTTA |  |
|  |  |  |  | [4] |
|  | nsP1 | 871R | ATGGAACACCGATGGTAGGTG |  |
|  |  |  |  |  |
| FG 2 | nsP1 | 616F | AACCCCGTTCATGTACAATGC |  |
|  |  |  |  |  |
|  | nsP1 | 1435R | CGGTACCACAAAGCTGTCAAAC |  |
|  |  |  |  |  |
| FG 3 | nsP1 | 1317F | CACTGACCTGCTGCTGTCTATG |  |
|  |  |  |  |  |
|  | nsP2 | 2130R | AGTCCTGCAGCTTCTTCCTTC |  |
|  |  |  |  |  |
| FG 4 | nsP1 | 1412F | CGAGTTTGACAGCTTTGTGGTA |  |
|  |  |  |  |  |
|  | nsP2 | 2227R | ATGACTGCAATTTTGTATGGGC |  |
|  |  |  |  |  |
| FG 5 | nsP2 | 1908F | CAATCTCGCCTGAAGACTTCC |  |
|  |  |  |  |  |
|  | nsP2 | 2709R | TCCACTACAATCGGCTTGTTG |  |
|  |  |  |  |  |
| FG 6 | nsP2 | 2530F | GTGCGGCTTCTTCAATATGATG |  |
|  |  |  |  |  |
|  | nsP2 | 3343R | TCCAGGCCTATTATCCCAGTG |  |
|  |  |  |  |  |
| FG 7 | nsP2 | 2577F | AACATCTGCACCCAAGTGTACC |  |
|  |  |  |  |  |
|  | nsP2 | 3504R | GTCTCCTGTTGGCCGGTATAAT |  |
|  |  |  |  |  |
| FG 8 | nsP2 | 3332F | TAATAGGCCTGGAGGGAAGATG |  |
|  |  |  |  |  |
|  | nsP3 | 4134R | CTACGCACTCTTCATCGTTCTT |  |
|  |  |  |  |  |
| FG 9 | nsP2 | 3885F | GAACGAGTCATCTGCGTATTGG |  |
|  |  |  |  |  |
|  | nsP3 | 4725R | ATATCTCTGCCATATCCACTGC |  |
|  |  |  |  |  |
| FG 10 | nsP3 | 4458F | TCTTTACAGCCATGGACTCGAC |  |
|  |  |  |  |  |
|  | nsP3 | 5273R | CGACAGGTACGGTGCTCATTAC |  |
|  |  |  |  |  |
| FG 11 | nsP3 | 5065F | TGTACAGGAAGCGAGTACGACC |  |
|  |  |  |  |  |
|  | nsP4 | 5874R | TCTACTTTGCGCGACTGATACC |  |
|  |  |  |  |  |
| FG 12 | nsP4 | 5630F | ACGGACGACGAGTTACGACTAG |  |
|  |  |  |  |  |

**S1 Table. Nucleotide sequences of conventional RT-PCR/sequencing primers for CHIKV.** Non-structural genes (NsP1, NsP2, NsP3 and NsP4).

The primer name indicates their position and direction on the nucleotide sequence of the S27 strain genome.
